# Metabolic syndrome increases risk for pulmonary embolism after hip and knee arthroplasty

**DOI:** 10.3325/cmj.2013.54.355

**Published:** 2013-08

**Authors:** Boris Mraovic, Brian R. Hipszer, Richard H. Epstein, Javad Parvizi, Edward C. Pequignot, Inna Chervoneva, Jeffery I. Joseph

**Affiliations:** 1 Department of Anesthesiology, Thomas Jefferson University Hospital, Philadelphia, PA, USA; 2 Rothman Institute of Orthopedics, Thomas Jefferson University Hospital, Philadelphia, PA, USA; 3 Department of Pharmacology and Experimental Therapeutics, Thomas Jefferson University, Philadelphia, PA, USA

## Abstract

**Aim:**

To investigate whether patients with metabolic syndrome (MetS) undergoing total hip or knee replacement have an increased risk for pulmonary embolism (PE).

**Methods:**

We studied patients undergoing total hip or total knee replacement from January 2001 to April 2006. The diagnosis of PE was based on a positive finding with a chest CT or a lung scan. Components of MetS were defined as 1) BMI≥30 kg/m^2^, 2) non-fasting preadmission glucose ≥11.1 mmol/L or diagnosis of diabetes, 3) hypertension, and 4) dyslipidemia. MetS was diagnosed if at least three of these components were present.

**Results:**

Of 7282 patients, 107 (1.47%) were diagnosed with PE. The incidence of PE in patients with 0, 1, 2, 3, and 4 MetS components was respectively 0.85% (16/1888; 95% confidence interval [CI] 0.5%-1.4%), 1.24% (31/2500; 95% CI 0.9%-1.8%), 1.76% (34/1936; 95% CI 1.2%-2.5%), 2.64% (21/796; 95% CI 1.7%-4.1%), and 3.09% (5/162; 95% CI 1.1%-7.4%). The independent risk factors for PE were age ≥70, knee as opposite to hip replacement, bilateral knee surgery, congestive heart failure, and MetS or the number of MetS components. The odds of PE independently increased 1.6 times (95% CI 1.01-2.56; *P* = 0.043) for patients with MetS and 1.23 times (95% CI 1.02-1.48; *P* = 0.028) per each additional MetS component.

**Conclusion:**

Patients with MetS are at increased risk for PE after total joint arthroplasty. The increasing number of MetS components significantly increased the incidence of PE.

Metabolic syndrome (MetS) is an increasing health problem, affecting 30% to 40% of the adult population in the USA (1). Its prevalence increases substantially with age. In patients older than 50 years, the prevalence is 44% (2) and increases in patients older than 60 years to 50% to 60% (3). With the increasing prevalence of MetS and an aging US population, it is likely that more patients with MetS will be undergoing major orthopedic surgeries such as total hip and knee arthroplasty.

Patients undergoing total hip and knee replacement surgeries are at risk of developing deep venous thrombosis (DVT) and pulmonary embolus (PE). Although thromboprophylaxis is routinely used after the surgery, the incidence of PE after hip and knee arthroplasty still remains high, between 0.5% and 10% (4-7). Manifestation of PE varies from asymptomatic to fatal. It may be expected that one out of 1000 patients undergoing hip and knee arthroplasty would die of PE (6,7).

MetS is defined as a cluster of risk factors for atherosclerosis. Several organizations proposed various criteria, but all include abnormal glucose metabolism, excessive body weight, elevated blood pressure, and abnormal lipid metabolism (8). The diagnosis of MetS is defined by the presence of 3 or more of these components. Although the syndrome is a constellation of factors that promote the development of atherosclerotic cardiovascular disease, recently it has been associated with venous thrombosis (9,10). A case-control study of patients who were referred to the thrombosis clinic in tertiary hospitals found that MetS was almost twice as common in patients diagnosed with idiopathic DVT than in those who did not have DVT (9). In patients with acute coronary syndrome or acute heart failure, the presence of MetS independently increased the risk of developing DVT by 2.38 (10). Our recent study showed that perioperative hyperglycemia >11.1 mg/dL and high body weight (BMI≥30 kg/m^2^) independently increased the risk of PE after hip and knee arthroplasty (11), but it is currently unknown whether patients with MetS have an increased risk for thromboembolic events after major orthopedic surgery. The aim of our study was to determine whether MetS and MetS components were predictors of symptomatic PE in patients undergoing total hip or knee replacement surgery.

## Materials and methods

This study was approved by Thomas Jefferson University Hospital Institutional Review Board. Electronic medical records of patients having undergone total hip or total knee replacement from January 1, 2001 to April 30, 2006 were retrospectively reviewed. We used the same data set as in our recently published study (11). In short, the study included patients who had undergone elective hip or knee arthroplasty, including revisions and bilateral procedures. In patients with multiple procedures during the study period, only the data from the most recent surgical procedure were included to avoid potential bias using the same patients with the same risk factors multiple times. The database was searched by ICD-9 codes for hip and knee arthroplasty (84.51, 81.53, 81.54, and 81.55) from January 1, 2001 to April 30, 2006. These records were matched with the Rothman Institute of Orthopedics database, which includes all postoperative complications experienced by each patient. Only laboratory data within 1 month prior the surgery were used for the analysis. Patients were excluded from analysis if they had (a) a diagnosis of paresis, paralysis, or cerebral palsy (55 patients); (b) a history of coagulopathy (22 patients); or (c) the preoperative placement of a Greenfield filter (23 patients).

The thromboembolic prophylaxis was with warfarin. Warfarin was started on the night of surgery, and the dose of the drug varied based on the perceived pharmacodynamics of the patients. In other words, elderly patients received lower starting doses (around 5 mg) than healthier and larger patients (10 mg). The additional dose was given depending on the response of the patient and it was titrated to achieve an International Normalized Ratio between 1.5 and 2.0. Patients also received 1000 IU of intravenous heparin at the time of hip dislocation during hip arthroplasty or prior to inflation of the tourniquet during knee arthroplasty. The heparin dosing was not weight-based as it has been shown that the lowest effective heparin dose at 1000 units accomplishes its antithrombotic effect without subjecting patients to potential risk of added bleeding (12). In the postoperative period, patients were subjected to early mobilization. All patients undergoing joint arthroplasty were ambulated early. Patients were required to get out of bed and try to walk within a few hours of surgery. They were seen at least twice by physical therapists who tried to walk the patients and encourage them to self-ambulate. Continuous passive-motion (CPM) devices were utilized following knee arthroplasties. They were used based on patients’ tolerance and preference, so the majority of the patients received between 4 to 5 hours of CPM.

Diagnosis of PE was made by computer tomography (CT) or lung VQ scan. During the study period, the imaging modality of choice for investigation of PE changed to more sensitive studies. Prior to May 2001, the modality for investigation of PE was lung VQ scan. It was replaced by a chest spiral CT study until November 2003, when it was upgraded to a multi-detector CT study. The imaging modality changes resulted in an increased detection of PE after hip and knee arthroplasty, as we previously reported (13). After May 2001, lung VQ scans were utilized only in patients with renal failure or allergy to intravenous contrast.

Characteristics of the study patients with and without PE and the test results of association between each potential risk factor and PE are presented in our previous study (11). In short, patients with PE were significantly older, had higher BMI, higher American Society of Anesthesiologists physical status, more frequently had primary unilateral or bilateral knee surgery, had longer duration of surgery, and more frequently had history of congestive heart failure and endocrinological disease than patients without PE (11).

A modified definition of MetS was adapted from The World Health Organization (WHO) and the National Cholesterol Education Program – Adult Treatment Program III (NCEP/ATP III) and applied to the data available in our databases (8). MetS components were defined as: 1) obesity – body mass index (BMI)≥30 kg/m^2^ (WHO definition), rather than waist circumference (NCEP/ATP III), since these data were not available; 2) dysglycemia – the glucose component included both glucose intolerance and non-fasting preadmission blood glucose ≥200 mmol/L (modified WHO), because patients were not instructed to fast before the preoperative visit, and/or diagnosis of diabetes mellitus (WHO and NCEP/ATP III); 3) elevated blood pressure – preoperative diagnosis of hypertension (WHO) was used because preoperative blood pressure measurements were not available; and 4) dyslipidemia – because of interchangeable use of a preoperative diagnosis of hyperlipidemia, hypercholesterolemia, and increased lipids for patients with elevated triglycerides, cholesterol and decreased HDL in our database, all patients with these diagnoses were included (modified WHO and NCEP/ATP III) (8). MetS was defined as the presence of three or more of the components described above.

### Statistics

Univariate association between the rate of PE incidence and MetS related covariates (MetS indicator, each MetS component, and the number of MetS components) was evaluated using Fisher exact and Mantel-Haenszel tests. Multivariate analysis of the PE incidence rate was performed using logistic regression with all independent covariates categorized. The initial model included all potential risk factors for PE considered in the previous study (11), not including the MetS components. Backward elimination was used to construct a parsimonious core model. To control for the change in increased sensitivity to detect PE over time, the year of surgery was always included as a covariate. Finally, the covariates related to MetS were added to this core model to evaluate the effect of MetS on PE while adjusting for other risk factors. This resulted in three final multivariate models, which included all covariates from the core model plus one of the following: (I) the MetS indicator (dichotomous); (II) the number of MetS components (0-4) as an ordinal covariate; (III) the four individual MetS components as separate predictors. This modeling approach of having a core model of the background variables associated with PE and then adding to it possible factors related to MetS allows comparing the predictive values of MetS-related covariates through the increase in the log-likelihood of models I-III as compared to the core model. Statistical analyses were performed using SAS 9.1.3 (SAS Institute Inc., Cary, NC; USA).

## Results

A total of 11 283 patients were reviewed. After exclusion of patients with missing data and the data from repeated admissions, 7282 patients who underwent elective, primary or revision, total hip, and knee replacements were analyzed. Patients were operated on average 15 ± 8 days after the preoperative clinic visit.

**Figure 1:**
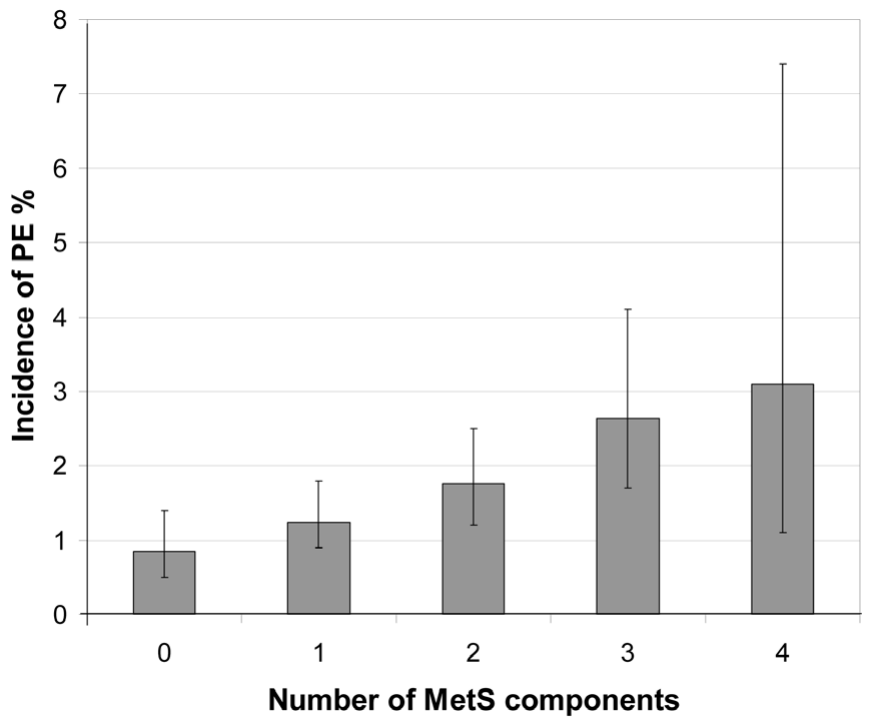
Incidence of pulmonary embolism (PE) by increasing number of metabolic syndrome (MetS) components. *The incidence of PE significantly increased with the number of MetS components, *P* < 0.001 (univariate Mantel-Haenszel test). Presented are PE incidence rates and 95% confidence intervals (CI). Each additional risk factor increased the odds for PE by 1.23 (95% CI 1.02-1.48), *P* = 0.028 (multivariate logistic regression).

The overall incidence of PE was 1.47% (107/7282 patients; 95% confidence interval [CI] 1.2%-1.8%). MetS was present in 13.2% (958/7282; 95% CI 12.4%-14.0%) of patients. In univariate analysis, patients with MetS had a significantly higher incidence of PE (2.7%, 26/958; 95% CI 1.8%-4.0%) than patients without MetS (1.3%, 81/6324; 95% CI 1.0%-1.6%) (*P* = 0.001) (Table 1). Among the four components defining MetS, only BMI≥30 kg/m^2^ and hypertension were univariately associated with a significant increase in the incidence of PE (Table 1). As the number of MetS components increased, so did the incidence of postoperative PE (*P* < 0.001, Figure 1). The incidence of PE in patients with 0, 1, 2, 3, and 4 MetS components was respectively 0.85% (16/1888; 95% CI 0.5% - 1.4%), 1.24% (31/2500; 95% CI 0.9%-1.8%), 1.76% (34/1936; 95% CI 1.2%-2.5%), 2.64% (21/796; 95% CI 1.7%-4.1%), and 3.09% (5/162; 95% CI 1.1%-7.4%) (Figure 1).

**Table 1:** Results of univariate analyses of metabolic syndrome and its components and the incidence of pulmonary embolism (PE) after major orthopedic surgery

		Number (%) of patients			
		with PE (N = 107)	without PE (N = 7175)	total (N = 7282)	P*
Metabolic syndrome	yes	26 (2.7)	932 (97.3)	958	0.001
	no	81 (1.3)	6243 (98.7)	6324	
Number of metabolic syndrome components	0	16 (0.85)	1872 (99.15)	1888	<0.003
	1	31 (1.24)	2469 (98.76)	2500	
	2	34 (1.76)	1902 (98.24)	1936	
	3	21 (2.64)	775 (97.36)	796	
	4	5 (3.09)	157 (96.91)	162	
Individual components of metabolic syndrome					
body mass index	<30 kg/m^2^	38 (0.9)	3966 (99.1)	4004	<0.001
	≥30 kg/m^2^	69 (2.1)	3209 (97.9)	3278	
dyslipidemia		26 (2.7)	932 (97.3)	958	0.169
hypertension		68 (1.8)	3806 (98.2)	3874	0.032
dysglycemia		19 (2.1)	867 (97.9)	886	0.099

*Mantel-Haenszel (number of components) or Fisher exact test.

In the multivariate core model not including MetS-related covariates, the increased risk of PE was significantly associated with age, knee as opposite to hip replacement, bilateral knee as opposite to unilateral knee replacement, and CHF (Table 2A). Year of surgery was included in the core model as described in Methods. During the study period, the incidence of diagnosed PE increased (13).

**Table 2:** Results of multivariate analyses of the incidence of pulmonary embolism after major orthopedic surgery. Part A – results from the core model not including the metabolic syndrome related covariates. Part B – results from three models combining the covariates from the core model and covariates related to metabolic syndrome: *dichotomous indicator of metabolic syndrome; †the number of metabolic syndrome components treated as ordinal covariate; ^‡^individual metabolic syndrome components considered simultaneously

Covariate/base comparison	Odds ratio (95% confidence interval)	*P*	Increase in log-likelihood
A			
Age (yrs)	Overall	0.007^§^	
50-60 /<50	1.43 (0.52- 3.90)	0.486	
60-70 /<50	1.80 (0.69 - 4.72)	0.232	
70-80 /<50	2.96 (1.15- 7.63)	0.025^§^	
>80 /<50	3.74 (1.33- 10.48)	0.012^§^	
Knee/hip	2.77 (1.73- 4.45)	<0.001^§^	
Bilateral knee/unilateral knee	2.19 (1.34- 3.57)	0.002^§^	
Congestive heart failure	3.12 (1.46- 6.68)	0.003^§^	
Year of surgery	Overall	0.051^§^	
2002/2001	2.02 (0.74 - 5.51)	0.170	
2003/2001	2.06 (0.82 - 5.17)	0.124	
2004/2001	3.41 (1.42 - 8.17)	0.006^§^	
2005-6/2001	2.64 (1.10 - 6.32)	0.030^§^	
B			
Metabolic syndrome*	1.61 (1.01 - 2.56)	0.043^§^	1.90
Number of metabolic syndrome components (0-4)^†^	1.23 (1.02 - 1.48)	0.028^§^	2.37
Hypertension^‡^	0.93 (0.61 - 1.42)	0.736	
Dyslipidemia^‡^	1.09 (0.68 - 1.72)	0.728	
Body mass index ≥30 kg/m^2‡^	2.19 (1.42 - 3.36)	<0.001^§^	
Dysglycemia^‡^	0.97 (0.57 - 1.65)	0.916	

^§^Statistically significant <0.05.

After adjusting for all other significant risk factors, patients with MetS still had 1.6 (95% CI 1.01-2.56; *P* = 0.043) times greater odds for developing PE than patients without MetS (Table 2B, model I). Considering the number of MetS components in the multivariate model controlling for other significant risk factors, each additional MetS component increased the odds for PE by 1.23 (95% CI 1.02-1.48; *P* = 0.028) (Table 2B, model II). Thus, patients with two MetS components had 1.51 (1.23^2^) times (95% CI 1.04-2.19) higher odds of PE than patients with no MetS components. The increase in the model log-likelihood (from the likelihood of the core model) was higher (2.37) when the ordinal number of MetS components was added to the core model than when the dichotomous MetS covariate was added (1.90) (Table 2B). This suggests that the number of MetS components may have better utility predicting PE than MetS as a dichotomous indicator.

When the four covariates defining MetS were added to the core multivariate model (model III), only BMI≥30 kg/m^2^ significantly increased the odds of PE, by 2.2 times (95% CI 1.4-3.4). This suggests that BMI might be the most important MetS component regarding the incidence of PE in this clinical setting.

## Discussion

This study showed that patients with MetS had increased risk of inhospital symptomatic PE after total hip and knee arthroplasty. The increasing number of MetS components significantly increased the incidence of PE. Overall, the risk of PE was increased by 23% for each additional component of MetS. The most important MetS component was BMI.

Our findings are consistent with three recent case-control studies evaluating MetS and the risk of venous thromboembolism (9,10,14). All three studies showed that elevated triglycerides significantly increased the risk for DVT (9,10,14), while our study showed that hyperlipidemia was not an independent risk factor for PE. This discrepancy could possibly be explained using our wider definition of dyslipidemia compared to the NCEP/ATP III criteria. Indeed, mean HDL and ongoing therapy with statins in Ageno’s study (9) and hypercholesterolemia and decreased HDL in Ambrosetti’s study (10) were not found to be associated with an increased risk for DVT. We are not aware of a study investigating the correlation between dyslipidemia and incidence of thromboembolic events after hip and knee arthroplasty in the current literature, although there is a growing body of evidence that dyslipidemia increases the risk of venous thromboembolism in the general population (15).

Also, our study is consistent with previous studies showing that obesity is a risk factor for PE after hip and knee arthroplasty (16,17). Ageno et al (9) and Ambrosetti et al (10) found that for the prediction of DVT MetS was independent of BMI, but the waist circumference was used as an obesity component of MetS. In the study by Ay et al (14), as well in this study, MetS was not independent of BMI.

All three MetS case-control studies found that increased fasting glucose was not a significant risk factor for DVT (9,10,14). Our patients were not instructed to fast before blood was sampled for preoperative testing, which precluded us from using fasting glucose level as a component of MS (WHO and NCEP/ATP III definition of MS) (8). In our previous study, hyperglycemic patients (blood glucose≥11.1 mmol/L) had high incidence of PE (5.1%) (11). It is unknown if more aggressive perioperative glycemic control influences the incidence for developing of PE after the surgery.

The MetS case-control studies did not find that high blood pressure increased the risk of DVT (9,10,14). In our study, the univariate analysis showed that hypertension increased the incidence of PE significantly, but the effect went away in the multivariate analysis. In support of this finding, hypertension also was not found to be a risk factor for PE after orthopedic surgery (16).

The possible mechanisms for thromboembolism in patients with MetS are not completely understood. This topic has been reviewed in detail elsewhere (18,19). In short, there is no agreement in regards to the initial metabolic abnormality. It is either obesity or insulin resistance/glucose intolerance. But there is concurrence that MetS is a proinflammatory and prothrombotic state. Obesity increases adipokines and hyperglycemia induces oxidative stress, while both can lead to activation of the inflammatory and coagulation cascade.

The limitations of this study are its retrospective data collection and missing baseline preoperative data. However, information regarding the outcome (PE) was complete and the missing data was spread evenly across the study period. We evaluated missing patients with PE and found no significant association between the incidence of PE and the missing data status (complete vs missing). Therefore, the results of the study may have not been skewed despite missing a large number of data. Over the study period, the mode of the detection of PE was changed. We have previously published a study that demonstrated increasing sensitivity of imaging modality resulting in a higher probability of a positive finding for PE (13). We incorporated year of the surgery in our analysis models to adjust for the change in the detection modality and the change over time in the other possible unforeseen variables. The change in the PE detection did not appear to influence out results since MetS and the number of MetS components remained independently significant predictors for PE. Another limitation is the modified definition of MetS. Our definition did not include patients who had borderline hypertension, insulin tolerance, impaired fasting glucose, elevated triglycerides, low HDL, and did not consider waist circumference, whereas these patients are included in the WHO and ATP III definitions of MetS. Indeed, the prevalence of MetS according to this modified definition was 13.2%, which was less than expected according to US population data (30%-40%) (1). Our modified definition probably included patients with more severe pathological conditions and diseases compared to WHO and ATP III definitions (8).

The American Academy of Orthopedic Surgeons recommended that chemical prophylaxis should be based on the risk stratification for PE and the risk of bleeding (*http://www.aaos.org/Research/guidelines/PE_guideline.pdf*). Data from our study suggest that patients receiving warfarin for thromboprophylaxis have a greater than standard risk for developing PE after hip and knee surgery, if they have MetS. Clinicians may use components of MetS to stratify the risk for PE after arthroplasty along with older age and history of CHF. The diagnosis of MetS or the presence of its components would alert an anesthesiologist of potentially aggressive thromboprophylaxis by the surgeon. This may influence the anesthesia and pain management choice, eg, if the patient is at greater risk for PE and more aggressive antithrombotic therapy is being considered, then perhaps the decision to place an epidural catheter should be carefully considered.

In summary, this study showed that patients with MetS had an increased risk of PE after hip and knee arthroplasty. Moreover, the incidence of PE correlated with the number of MetS components. The increasing number of MetS components significantly increased the incidence of PE. A prospective study is needed to determine if better preoperative control of MetS components decreases the incidence of PE after hip and knee total replacement surgery.
